# Preoperative Serum Prealbumin Level and Adverse Prognosis in Patients With Hepatocellular Carcinoma After Hepatectomy: A Meta-Analysis

**DOI:** 10.3389/fonc.2021.775425

**Published:** 2021-10-21

**Authors:** Yu Fan, Yimeng Sun, Changfeng Man, Yakun Lang

**Affiliations:** Institute of Molecular Biology & Translational Medicine, The Affiliated People’s Hospital, Jiangsu University, Zhenjiang, China

**Keywords:** prealbumin, hepatocellular carcinoma, hepatectomy, overall survival, hepatic insufficiency, meta-analysis

## Abstract

**Background:**

Prealbumin is a sensitive indicator of liver function and nutritional status.

**Objectives:**

This meta-analysis aimed to examine the association of the serum prealbumin level with the prognosis of patients with hepatocellular carcinoma (HCC) undergoing hepatectomy.

**Methods:**

We comprehensively searched the PubMed, Embase, Wanfang, China Academic Journals (CNKI), and SinoMed databases up to September 1, 2021. Eligible studies should report the association of the serum prealbumin level with prognosis and provide the multivariable-adjusted risk estimates of the outcomes of interest in HCC patients undergoing hepatectomy.

**Results:**

A total of 11 studies with 7,442 HCC patients were identified and analyzed. Meta-analysis of a fixed effects model showed that a low serum prealbumin level was associated with poor overall survival [hazard ratio (HR) = 1.54, 95% confidence interval (CI) = 1.42–1.68], recurrence-free survival (HR = 1.34, 95% CI = 1.17–1.52), and a higher risk of postoperative hepatic insufficiency (HR = 2.21; 95% CI = 1.36–3.60) in HCC patients. Sensitivity and subgroup analyses confirmed the robustness of low serum prealbumin in predicting poor overall survival.

**Conclusions:**

This meta-analysis indicated that a low preoperative serum prealbumin level was significantly associated with adverse prognosis in HCC patients undergoing hepatectomy.

## Introduction

Hepatocellular carcinoma (HCC) is the principal type of primary liver cancer in adults and accounts for approximately 90% of liver malignancy (1). Despite the advances in treatment approaches for HCC, it remains the second leading cause of cancer-related mortality because of its distant metastasis and tumor recurrence (2). The 5-year survival of HCC is about 10%–20% (3, 4). Hepatectomy is the main treatment for HCC (5). However, only one-third of patients with early-stage HCC could receive surgical resection or liver transplantation due to advanced stage of disease or cirrhosis-related hepatic insufficiency. Therefore, the prognostic assessment of HCC patients before surgery is an unmet demand.

Prealbumin, also known as transthyretin, is a homotetrameric protein synthesized by the liver (6). Serum prealbumin level is a sensitive indicator of liver function and nutritional status. Prealbumin has a short biological half-life and reflects recent status, rather in contrast to albumin (7). Thus, prealbumin is a better indicator of liver function and nutritional status (8). Several studies (9–15) have linked low serum prealbumin levels with adverse outcomes in HCC patients after hepatectomy. However, conflicting results have been obtained regarding the association of preoperative prealbumin level with overall survival (OS) (16, 17). Nevertheless, the magnitude of the reported risk estimates considerably varies among studies.

A previous meta-analysis involving 3,470 patients has evaluated the prognostic value of prealbumin in liver cancer (18). However, this well-designed meta-analysis enrolled heterogenous patient populations, including those undergoing chemotherapy and molecular targeted therapy. To address these knowledge gaps, we performed a more focused meta-analysis in the current study to assess the association of preoperative prealbumin level with adverse outcomes in HCC patients undergoing hepatectomy.

## Materials and Methods

### Data Sources and Literature Search

This meta-analysis was conducted according to the Preferred Reporting Items for Systematic Reviews and Meta-Analyses guidelines (19). Two independent authors comprehensively searched PubMed, Embase, Wanfang, China Academic Journals (CNKI), and SinoMed databases up to September 1, 2021. The keywords included: “prealbumin” OR “transthyretin” AND “hepatocellular carcinoma” OR “hepatocellular cancer” OR “liver cancer.” The reference lists of related studies were also manually reviewed for additional studies.

### Study Selection

The inclusion criteria were as follows: 1) population: patients with HCC undergoing hepatectomy; 2) exposure: serum prealbumin level before surgery; 3) comparison: patients with a lower prealbumin level *versus* those with a higher prealbumin level; outcome measures: overall survival, recurrence-free survival, and postoperative hepatic insufficiency; 5) study design: prospective or retrospective cohort studies; and 6) reported multivariable adjusted risk summary for the outcomes of interest according to the prealbumin category. The following exclusion criteria were used: 1) patients with a specific type of HCC; 2) patients who did not receive surgery; 3) risk estimates reported using univariate analysis; and 4) studies with overlapping patients.

### Data Extraction and Quality Assessment

The extracted data included the following: surname of the first author, publication year, country of origin, study design, number of patients, percentage of male gender, mean/median age, cutoff value of low prealbumin, outcome measures, fully adjusted risk estimate, length of follow-up, and adjustment for confounders. The methodological quality of the eligible studies was evaluated with the Newcastle–Ottawa Scale (NOS) (20). A study with a score of 7 points or over was classified as high quality. Two independent authors conducted the data extraction and quality assessment. Any disagreement between these processes was resolved by mutual consent.

### Statistical Analysis

All meta-analyses were performed using the STATA 12.0 (STATA Corp LP, College Station, TX, USA). The impact of the preoperative serum prealbumin level on adverse outcomes was estimated by pooling the multivariable-adjusted hazard ratio (HR) and their 95% confidence interval (CI) for the lowest *versus* the highest prealbumin category. Heterogeneity was evaluated using the Cochrane *Q* test and *I* ^2^ statistic. A *p-*value of <0.10 of the Cochrane *Q* test or *I* ^2^ statistic ≥50% indicated the presence of significant heterogeneity, and a random effects model was selected to pool the risk summary. Otherwise, we selected a fixed effects model if heterogeneity was not found. Leave-one-out study sensitivity analysis was conducted to investigate the stability of the pooling results. To identify potential sources of heterogeneity across studies, subgroup analyses were performed according to sample size, study design, prealbumin cutoff value, follow-up duration, and NOS points. Publication bias was examined using Begg’s test (21) and Egger’s test (22).

## Results

### Search Results and Study Characteristics

**Figure 1:**
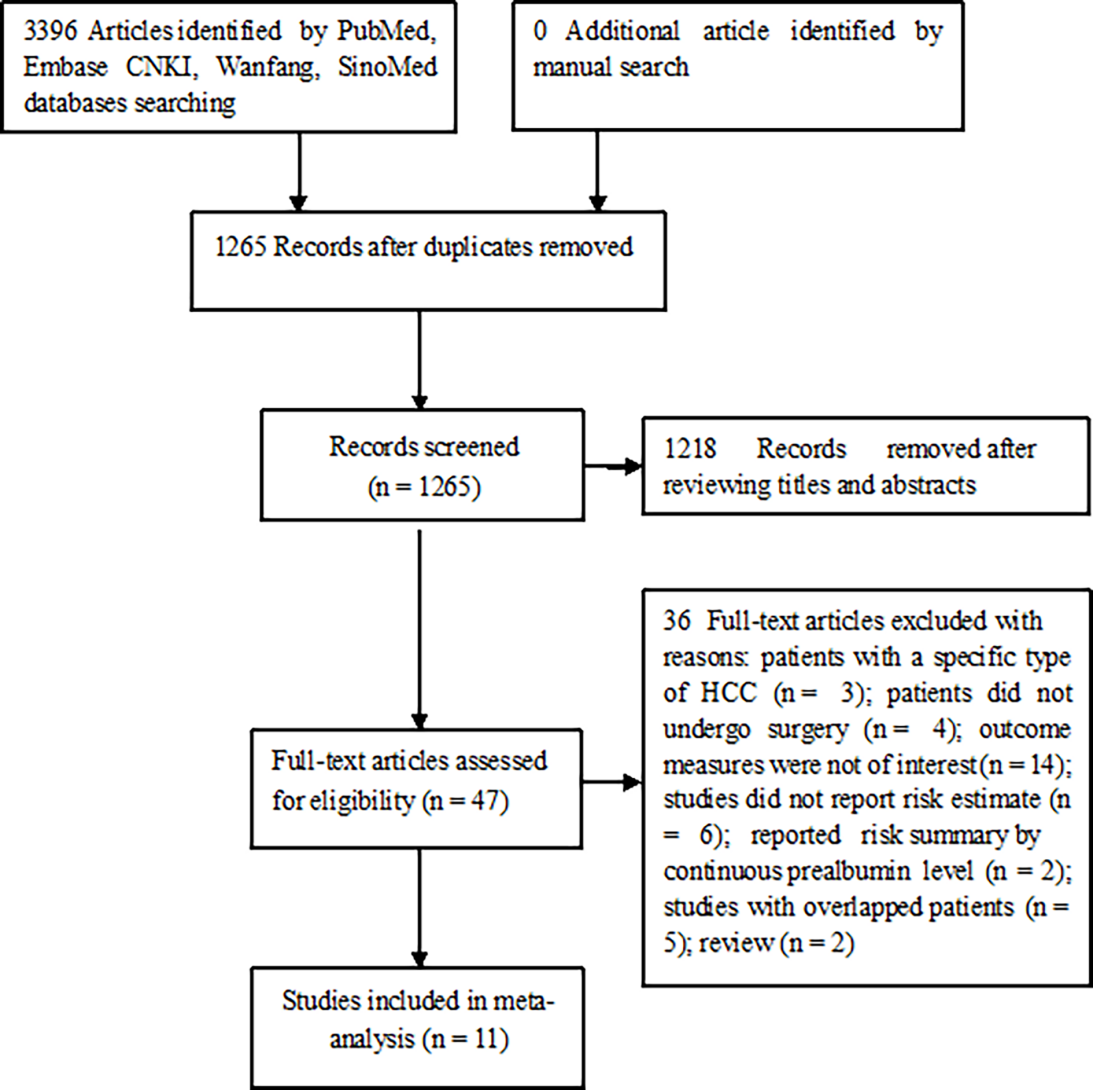
Flowchart of the study selection process.

Our literature search identified 1,265 unique publications. Among them, 1218 were scanned for their titles or abstracts and 47 were retrieved for full-text evaluation. After applying the predefined selection criteria, 11 studies (9–14, 16, 17, 23–25) satisfying our inclusion criteria were finally included in this meta-analysis (**Figure 1**).

**Table 1** describes the baseline characteristics of the included studies. A total of 11 studies with 7,442 HCC patients were identified and analyzed. These studies were published from 2012 to 2021. One study (11) was performed in Japan, and others were conducted in China. Two studies (12, 13) adopted a prospective design, and others were retrospective studies. The reported mean/median age of patients ranged between 49.5 and 69.6 years. The mean/median follow-up duration was from 21 days to 67.7 months. Seven studies (11, 12, 14, 17, 23–25) reported the distribution of Child–Pugh classes. Approximately 90% of patients were grouped into Child–Pugh class A, and only one study (11) included the Child–Pugh class C. Substantial Child–Pugh class A patients also had a low prealbumin value (14, 23–25). Compared with the normal prealbumin group, the low prealbumin group had a significantly higher incidence of Child–Pugh grade B (14, 23, 25). Regarding the methodological quality, all included studies were considered to be of high quality, with NOS scores ranging from 7 to 8 points.

**Table 1:** Main characteristics of individual studies.

First author, year	Country	Study design	Sample size (% male)	Mean/median age (years)	Child–Pugh class	Low albumin	Prealbumin cutoff (mg/dl)	Outcome measures: HR or RR (95% CI)	Adjustment for covariates	Follow-up duration	Total NOS
Huang et al. (9)	China	R	427 (84.8)	51.1 ± 10.4	NR	NR	<17 *vs*. ≥17	HI: 3.19 (1.19–8.60)	Multivariate Cox proportional hazard analysis	21 days	7
Zhao et al. (10)	China	R	373 (87.9)	52 (25–81)	NR	NR	≤15.2 *vs*. >15.2	OS: 1.45 (1.03–2.05) RFS: 1.56 (1.18–2.07)	Multivariate Cox proportional hazard analysis	60 months	7
Shimura et al. (11)	Japan	R	25 (88)	69.6 (55–84)	A: 88%; B: 8%; C: 4%	NR	≤11.4 *vs*. >11.4	OS: 4.84 (1.12–20.9)	Multivariate Cox proportional hazard analysis	67.7 months	7
Wen et al. (12)	China	P	613 (81.9)	52.9 ± 10.4	A: 88.9%; B: 10.1%	10.9%	≤12 *vs*. >19 (men); ≤11 *vs*. >17 (women)	OS: 1.37 (1.13–1.65)	Multivariate Cox proportional hazard analysis	23 months	7
Zhang et al. (13)	China	P	230 (83.9)	51.6 ± 12.2	NR	NR	≤15.3 *vs*. >15.3	OS: 2.35 (1.25–4.39)	Age, sex, alcohol, tobacco, hypertension, diabetes, chemotherapy, tumor size, tumor number, differentiation, BCLC stage, AFP	>36 months	7
Jia, 2019 (14)	China	R	526 (85.6)	NR	A: 94.9%; B: 5.1%	10.3%	≤18.2 *vs*. >18.2	OS: 1.64 (1.27–2.12)	Age, sex, tumor size, tumor number, tumor capsule, albumin, ALT, TB, AFP, AST, macrovascular invasion, cirrhosis, HBsAg, Child–Pugh, BCLC stage	56 months	7
Li et al. (23)	China	R	1,483 (89)	51 ± 11	A: 90%; B: 10%	NR	≤17 *vs*. >17	OS: 1.45 (1.24–1.70) RFS: 1.28 (1.10–1.48)	Comorbid illness, ECOG performance status, cirrhosis, portal hypertension, TB, AST, albumin, AFP, maximum tumor size, tumor number, vascular invasion, satellites, tumor differentiation, blood loss, blood transfusion, major hepatectomy	67 months	8
Li et al. (24)	China	R	2,022 (86.0)	49.5 ± 11.2	A: 90.1%; B: 9.9%	10.7%	≤16.6 *vs*. >16.6	OS: 1.69 (1.44–1.98)	Age, sex, tumor number, tumor size, tumor capsule, HBsAg, cirrhosis, AFP, ALB, AST, ALT, TB, Child–Pugh, BCLC stage	37.4 months	8
Wang, 2020 (16)	China	R	142 (82.4)	NR	NR	NR	≤20 *vs*. >20	OS:1.45 (0.96–2.17)	Tumor diameter, tumor number, HBsAg, cirrhosis, C-reactive protein, CNLC stage	60 months	7
Li et al. (25)	China	R	1,356 (88.9)	50.6 ± 10.6	A: 90.4%; B: 9.6%	NR	<17 *vs*. ≥17	OS: 2.50 (1.22–5.15) HI: 1.97 (1.12–3.43)	Sex, comorbid illness, platelets, AST, tumor size, tumor number, vascular invasion, blood loss, blood transfusion, extent of hepatectomy, type of resection, operation time	3 months	8
Xu et al. (17)	China	R	245 (88.2)	Not reported	A: 93.5%; B: 6.5%	8.98%	≤20 *vs*. >20	OS: 1.43 (0.66–1.78)	Multivariate Cox proportional hazard analysis	60 months	7

HR, hazard ratio; RR, risk ratio; CI, confidence interval; P, prospective; R, retrospective; NR, not reported; HCC, hepatocellular carcinoma; OS, overall survival; RFS, recurrence-free survival; HI, hepatic insufficiency; ALT, alanine transaminase; AST, aspartate aminotransferase; BCLC, Barcelona Clinic Liver Cancer; HBsAg, hepatitis B surface antigen; TB, total bilirubin; ECOG, Eastern Cooperative Oncology Group; AFP, Alpha fetoprotein; CNLC, China Liver Cancer; NOS, Newcastle–Ottawa Scale.

### Overall Survival

**Figure 2:**
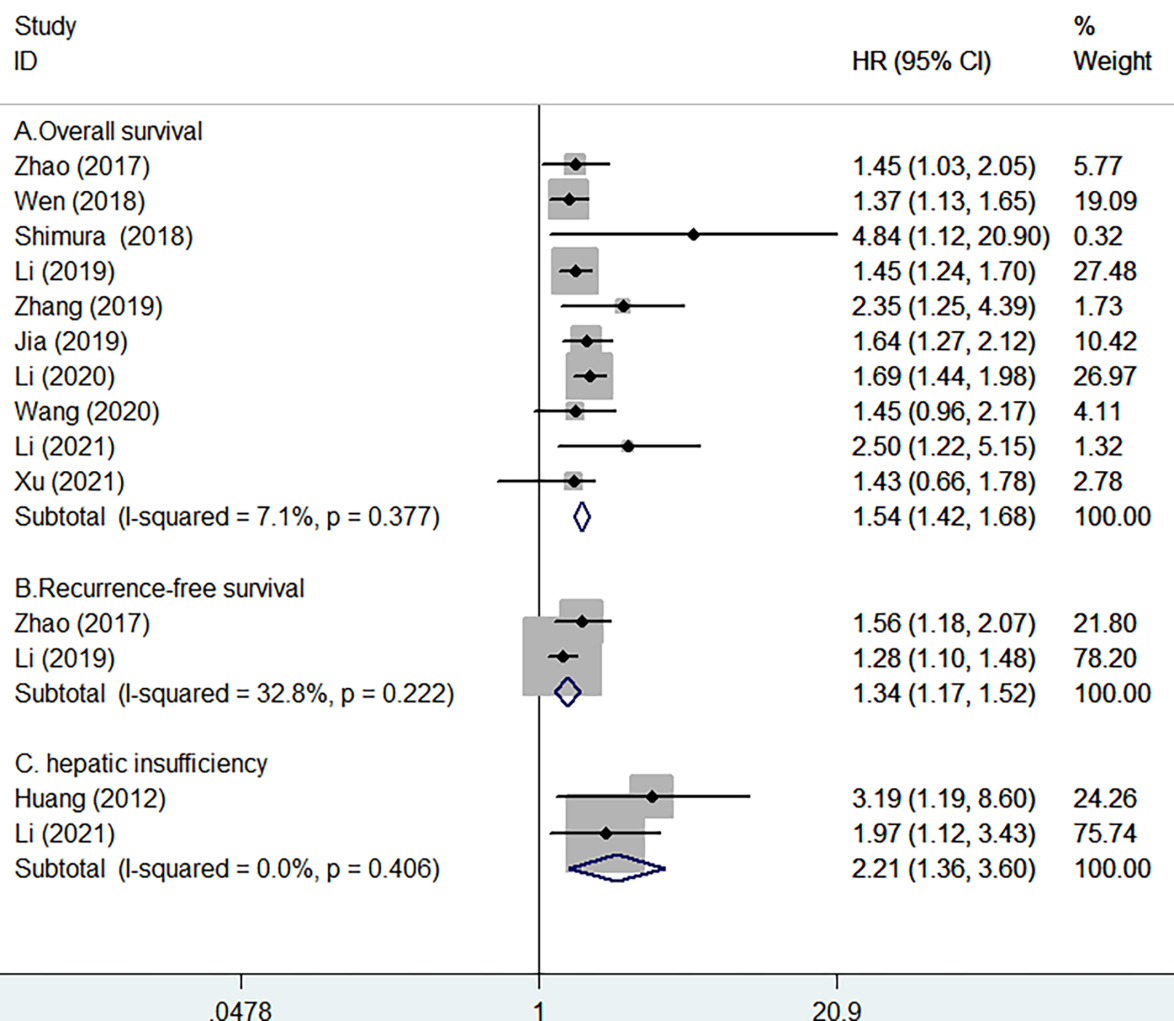
Forest plots showing the pooled hazard ratios (HR) and 95% CI of overall survival **(A)**, recurrence-free survival **(B)**, and postoperative hepatic insufficiency **(C)** for lower *versus* higher serum prealbumin levels.

**Figure 3:**
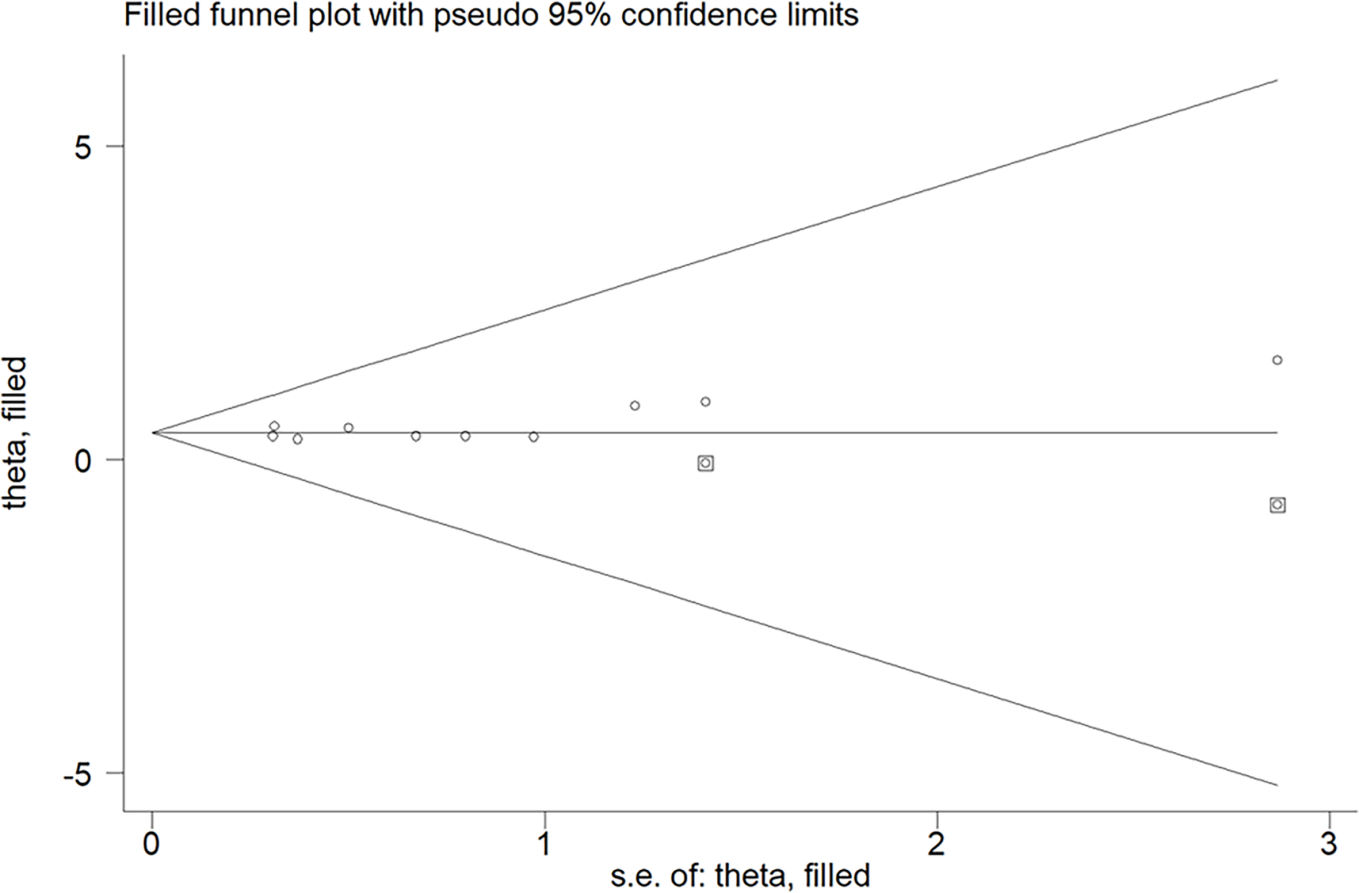
Funnel plot showing the impact of a lower prealbumin level on overall survival. The *circles alone* are real studies; *circles enclosed in boxes* are “filled” studies.

Ten studies (10–14, 16, 17, 23–25) provided data on the value of prealbumin level in predicting OS. As shown in **Figure 2A**, no significant heterogeneity (*I* ^2^ = 7.1%, *p* = 0.377) was observed. The meta-analysis indicated that a higher prealbumin level was associated with poorer OS (HR = 1.54, 95% CI = 1.42–1.68) than was a lower prealbumin level in the fixed effects model. Leave-one-out study sensitivity analysis suggested that the pooled risk estimates were statistically significant (data not shown). Additionally, the predictive value of a low prealbumin level showed no significant alterations in the different sample sizes, study designs, prealbumin cutoff values, and the follow-up duration subgroups (**Table 2**). However, Begg’s test (*p* = 0.032) and Egger’s test (*p* = 0.099) indicated clear evidence of publication bias. After imputing two potential missing studies, the pooled HR of OS was 1.53 (95% CI = 1.12–2.09) under trim-and-fill analysis (**Figure 3**).

**Table 2:** Results of subgroup analysis on overall survival.

Subgroup	No. of studies	Pooled hazard ratio	95% confidence interval	Heterogeneity between studies
Study design				
Prospective	2	1.43	1.20–1.72	*p* = 0.107, *I* ^2^ = 61.5%
Retrospective	8	1.57	1.43–1.73	*p* = 0.508, *I* ^2^ = 0.0%
Sample size				
<1,000	7	1.50	1.32–1.69	*p* = 0.438, *I* ^2^ = 0.0%
≥1,000	3	1.58	1.42–1.77	*p* = 0.184, *I* ^2^ = 40.9%
Prealbumin cutoff				
≤18 mg/dl	7	1.54	1.41–1.69	*p* = 0.158, *I* ^2^ = 35.4%
>18 mg/dl	3	1.56	1.28–1.90	*p* = 0.824, *I* ^2^ = 0.0%
Follow-up duration				
>36 months	8	1.58	1.44–1.73	*p* = 0.669, *I* ^2^ = 0.0%
≤36 months	2	1.42	1.19–1.71	*p* = 0.113, *I* ^2^ = 60.1%

### Recurrence-Free Survival

Two studies (10, 23) provided data on the value of prealbumin level in predicting RFS. **Figure 2B** shows no significant heterogeneity (*I* ^2^ = 32.8%, *p* = 0.222) between studies. The pooled HR of RFS was 1.34 (95% CI = 1.17–1.52) for the higher *versus* lower prealbumin level in the fixed effects model.

### Hepatic Insufficiency

Two studies (9, 25) provided data on the value of prealbumin level in predicting postoperative hepatic insufficiency. As shown in **Figure 2C**, there was no significant heterogeneity (*I* ^2^ = 0.0%, *p* = 0.406) between studies. The pooled HR of hepatic insufficiency was 2.21 (95% CI = 1.36–3.60) for the higher *versus* lower prealbumin level in the fixed effects model.

## Discussion

The current meta-analysis suggested that a low preoperative serum prealbumin level was independently associated with poor OS and RFS, as well as increased risk of postoperative hepatic insufficiency, in HCC patients undergoing hepatectomy. HCC patients with a low serum prealbumin level after hepatectomy had approximately 54% and 34% reduced risks of OS and RFS, respectively. Moreover, a low serum prealbumin level was associated with a 2.21-fold higher risk of postoperative hepatic insufficiency. These findings indicated that the preoperative serum prealbumin level may serve as a promising predictor of adverse outcomes in HCC patients.

Analysis of the serum prealbumin level using continuous variables also supported its predictive value. A decrease of 0.1 g/L prealbumin level increased the odds ratio of postoperative liver function insufficiency to 3.91 (15). Per standard deviation increase in the prealbumin level was associated with a 23% lower risk of mortality in HCC patients after hepatectomy (26). Our subgroup analysis further indicated that prediction of OS risk using the low prealbumin level appeared to be more pronounced in studies with a follow-up of >36 months than in those with ≤36 months of follow-up. This finding suggested that the impact of a low prealbumin level on OS tended to be stronger with increased duration of follow-up.

The serum albumin and prealbumin levels can be used to reflect the protein nutritional status, inflammatory state, and hepatic protein synthesis capability. Serum albumin level is more commonly used in clinical practice than is the prealbumin level. However, serum albumin level is often affected by renal function, hydration, and exogenous supplement of albumin (27). There is skepticism in using albumin as a nutritional marker because of its lack of specificity and long half-life of 20 days (28). Serum prealbumin is recommended as a nutritional biomarker, particularly in the elderly population (29). The major advantage of prealbumin as a nutritional biomarker is its short half-life of 2–3 days and its high specificity and sensitivity in the assessment of hepatic functional reserve (30, 31). Moreover, the serum prealbumin level is unaffected by intestinal protein losses (32). Therefore, prealbumin level is a more reliable and faster indicator for assessing a patient’s nutritional level. In the multivariable analysis, the preoperative prealbumin level independently predicted OS, whereas the albumin level lost its statistical significance (11, 12, 23, 25). These findings indicated that the serum prealbumin level may have better predictive value than does the albumin level in HCC patients. Notably, the above findings should be interpreted with caution due to the small number of studies included.

Our meta-analysis highlighted that the determination of preoperative prealbumin levels can improve the risk stratification of HCC patients. The identification of HCC patients with a low prealbumin level may help clinicians estimate the liver function and nutritional status. HCC patients with a low prealbumin level should receive close monitoring and active nutritional support.

The current meta-analysis has several limitations. Firstly, selection bias may have occurred because of the retrospective nature of most eligible studies. Secondly, single determination of the prealbumin level rather than a dynamic measurement may have resulted in the misclassification of patients into categories. Thirdly, various cutoff values for low level of serum prealbumin were reported in the included studies, conferring difficulty for clinical applications. Future studies should further establish the optimal cutoff value for low level of prealbumin. Fourthly, due to insufficient data, we failed to perform subgroup analysis according to the clinicopathologic data, including cirrhosis, C-reactive protein, Barcelona Clinic Liver Cancer stage, or the alpha fetoprotein level. Fifthly, the serum prealbumin level may be affected by obstructive jaundice, hyperthyroidism, nephritic syndrome, or ulcerative colitis. Particularly, not all included studies adjusted for the tumor factors and cirrhosis in their statistical models. The lack of adjustment for these important confounders may have led to the overestimation of the predictive value of prealbumin. Finally, apart from one study (11) originating from Japan, all the included studies were from China, where there is a predominant hepatitis B virus endemic area, thereby restricting the generalizability of our study to the West.

## Conclusion

A low preoperative serum prealbumin level is possibly an independent predictor of poor survival and postoperative hepatic insufficiency in HCC patients undergoing hepatectomy. The serum prealbumin level may be used for the risk stratification of HCC patients. However, the current findings should be interpreted with caution due to the retrospective nature of most of the eligible studies.

## Data Availability Statement

The original contributions presented in the study are included in the article/supplementary material. Further inquiries can be directed to the corresponding authors.

## Author Contributions

CM and YL contributed to the study and guaranteed the integrity of study. YF and YS searched the literature, extracted the data, assessed the study quality, and conducted the statistical analysis. YF wrote the manuscript. YL revised/edited the manuscript. All authors contributed to the article and approved the submitted version.

## Funding

This work is supported by: 1) Jiangsu Innovative Team Leading Talent Fund (CXTDC2016006); 2) Jiangsu 333 Talent Fund (BRA2020016); 3) Suqian Science and Technology Support Project Fund (S201907); 4) Jiangsu Provincial Key Research and Development Special Fund (BE2015666); 5) Jiangsu Six High Peak Talent Fund (WSW-205); and 6) Zhenjiang Key Research and Development Fund (SH2021038).

## Conflict of Interest

The authors declare that the research was conducted in the absence of any commercial or financial relationships that could be construed as a potential conflict of interest.

## Publisher’s Note

All claims expressed in this article are solely those of the authors and do not necessarily represent those of their affiliated organizations, or those of the publisher, the editors and the reviewers. Any product that may be evaluated in this article, or claim that may be made by its manufacturer, is not guaranteed or endorsed by the publisher.
